# Mapping *C. difficile* TcdB interactions with host cell-surface and intracellular factors using proximity-dependent biotinylation labeling

**DOI:** 10.1128/mbio.03336-24

**Published:** 2025-01-17

**Authors:** Jennifer S. Ward, Karl J. Schreiber, John Tam, Ji-Young Youn, Roman A. Melnyk

**Affiliations:** 1 Department of Biochemistry, University of Toronto, Toronto, Ontario, Canada; 2 Molecular Medicine Program, The Hospital for Sick Children Research Institute, Toronto, Ontario, Canada; 3 Department of Molecular Genetics, University of Toronto, Toronto, Ontario, Canada; University of Oklahoma Health Sciences Center, Oklahoma City, Oklahoma, USA

**Keywords:** toxin, BioID, *C. difficile*, diphtheria toxin, receptor, biotin

## Abstract

Many bacterial toxins exert their cytotoxic effects by enzymatically inactivating one or more cytosolic targets in host cells. To reach their intracellular targets, these toxins possess functional domains or subdomains that interact with and exploit various host factors and biological processes. Despite great progress in identifying many of the key host factors involved in the uptake of toxins, significant knowledge gaps remain as to how partially characterized and newly discovered microbial toxins exploit host factors or processes to intoxicate target cells. Proximity-dependent biotinylation (e.g., BioID) is a powerful method to identify nearby host factors in living cells, offering the potential to identify host targets of microbial toxins. Here, we used BioID to interrogate proximal interactors of the multi-domain *Clostridioides difficile* TcdB toxin. Expressed fusions of TurboID to different fragments of TcdB identified several high-confidence proteins in the cytosol, including members of the Rho GTPase signaling network and the actin cytoskeletal network. Additionally, we developed an extracellular proximity labeling method using recombinant TurboID-toxin chimeras, which uncovered a limited number of cell-surface targets including LRP1, which was previously identified as a cell-surface receptor of TcdB. Our work reveals surface receptors and intracellular components exploited by bacterial toxins, highlighting key vulnerabilities in host cells.

**IMPORTANCE:**

Bacterial toxins are the causative agents of many human diseases. Further characterizing the intoxication mechanisms of these proteins is important for the development of vaccines and treatments for toxin-mediated disease. Proximity-dependent biotinylation approaches offer an orthogonal approach to complement genetic screens. Here, we evaluate the potential of this method to identify host-toxin interactions on the cell surface and in the cytosol, where the toxin modifies essential host targets. Critically, we have highlighted several limitations of this method as applied to protein toxins, which are important for researchers to weigh when considering this technique for exotoxin studies.

## INTRODUCTION

Exotoxins are multidomain proteins secreted by bacteria that are responsible for the devastating symptoms of many infectious diseases (1). While vaccination programs have effectively mitigated the harm of toxins such as Diphtheria and Tetanus, many other toxins lack vaccines or effective treatment options (2). These include members of the large clostridial toxin (LCT) family, which are responsible for illnesses ranging from acute diarrhea to deadly gynecological infections (3). To advance our understanding of toxins and generate treatments for toxin-mediated diseases, it is important to unravel the ways toxins interacts with host cells and impair cellular function.

Toxins with intracellular targets interact with specific host factors and exploit cellular processes to enter cells and enzymatically modify their host targets. There remain many uncharacterized features about known toxins that have already been identified, such as the full suite of host cell receptors they utilize and the action of the toxin intracellularly (1). Additionally, advances in bacterial genomics have revealed a multitude of putative toxins for which the host cell receptors and targets are unknown (4, 5). Early work in the 1960s and 1970s to probe toxin receptors and cellular targets made use of arduous and hazardous radiolabeling techniques wherein the protein toxin or host cell factors hypothesized to interact with the toxin were radiolabeled (6, 7). Later, genetic screens with cDNA libraries and chemical mutagenesis screens were used to identify receptors including diphtheria toxin (DT) receptor (8) and the anthrax toxin receptor (9). In recent years, sophisticated though labor-intensive screens such as microRNA-adapted short hairpin RNA (shRNA) libraries (10), gene-trap insertional mutagenesis screens (11), genome-wide siRNA screens (12), and CRISPR-Cas9 loss-of-function screens have been used to identify receptors and intracellular targets for exotoxins (10–13). Though powerful, these tools are limited in their ability to capture genes that are essential for host viability or host factors that are compensated for by redundant cellular processes. Additionally, these screens may miss key protein-protein interactions that are important but not essential for intoxication.

Orthogonal methods are needed to complement existing genetic approaches and fully elucidate the interaction landscape of toxin-host interactions and toxin localization. Proximity-dependent biotinylation techniques, a set of relatively new approaches to map proximal protein-protein interactions, could fill this role (14–16). In a proximity labeling screen, a protein of interest (“bait”) is expressed in actively growing cells as a genetic fusion to an abortive biotin ligase enzyme (BirA* from *Escherichia coli* [17]) or peroxidase-derived enzyme, APEX. These enzymes generate activated biotin moieties, which react with surface-exposed lysine (BirA*) or tyrosine (APEX) residues within the ~10–20 nm range (14, 18). Proximity labeling offers several advantages. First, as biotinylation occurs in living cells, it detects proximal interactions in the context of native subcellular environments. This contrasts with traditional affinity-purification methods that detect physical associations sustained throughout cell lysis and purification steps, where subcellular contexts are lost. Second, the covalent modification allows lysis and purification of proximal interactors in stringent buffer conditions, allowing extraction of proteins in highly insoluble compartments, including membrane-associated compartments or cytoskeletal structures. Labeled proteins from lysed cells are purified with streptavidin beads and identified with mass spectrometry. For BioID, which employs the biotin ligase BirA* (17), molecularly evolved versions called TurboID and miniTurbo (19) offer increased labeling kinetics and efficiency.

Enzyme-catalyzed proximity labeling with BioID or TurboID has been used extensively to capture endogenous mammalian protein-protein interactions and provide information about the spatial organization of proteins in the cell (20–25). BioID has also successfully been applied to the study of host-pathogen interactions. A recent study engineered a coronaviral replication/transcription complex bait protein to identify host protein interactions with the viral replication machinery (26). D’Costa et al. demonstrated the value of complementary BioID and immunoprecipitation-mass spectrometry screens to understanding bacterial effector-host interactions with their investigation of *Salmonella* type 3 secreted effectors (27). Applying traditional BioID to bacterial exotoxins that translocate themselves into cells to reach their intracellular targets, presents several challenges. Notably, this class of toxins interact with host targets in at least three different compartments/environments during their intoxication pathway: (i) at the cell surface, where toxins recognize and bind cell surface receptors; (ii) within acidified endosomes where they are often activated by host proteases; and finally (iii) within the cytosol where the catalytic moieties of toxins may encounter host chaperones to help refold before interacting with and enzymatically inactivating their cognate intracellular host targets. Each of these compartments requires specialized experimental conditions to allow both the “bait” and corresponding biotin ligase to function optimally. In addition, the highly cytotoxic nature of the catalytic domains requires the introduction of carefully considered mutations to the active site that minimally affect the host target repertoire.

**Fig 1:**
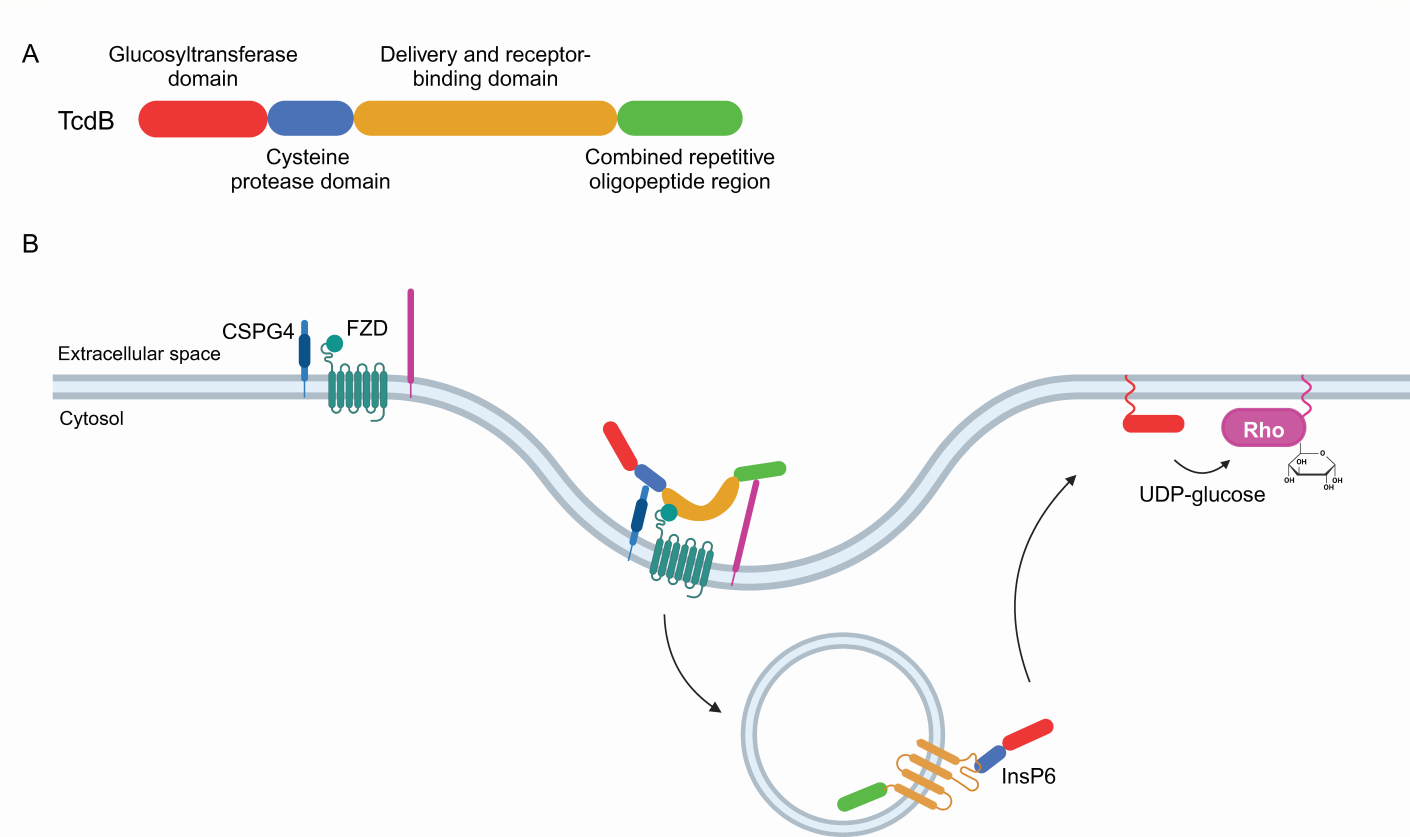
*C. difficile* toxin B (TcdB). (**A**) Domain architecture of TcdB. The N-terminal GTD is shown in red, followed by the CPD in blue, the DRBD in orange, and the CROP region in green. (**B**) TcdB binds multiple cell surface receptors (CSPG4, Frizzled [FZD]) and is internalized into early endosomes. The DRBD forms a pore in response to endosomal acidification, delivering the GTD and CPD to the cytosol. Once cleaved, the GTD docks to the inner plasma membrane and glucosylates Rho family small GTPases. Illustrations created on Biorender.com.

We present the first application of proximity labeling using an exogenously added recombinant protein that acts on and infiltrates cells—in this case, the translocating exotoxin, *Clostridioides difficile* toxin B (TcdB). Extensive studies over the past two decades have shown that TcdB and TcdA, two toxins produced by *C. difficile*, are the causative agents of disease. Based on several lines of evidence, TcdB appears to be the primary virulence determinant of disease (28–30). TcdB is a large single polypeptide of 2,366-amino acids with four functional domains: an N-terminal glucosyltransferase domain (GTD), a cysteine protease domain (CPD), a delivery and receptor-binding domain (DRBD), and a C-terminal combined repetitive oligopeptide (CROP) region (Fig. 1). After binding to one of its cell-surface receptors, TcdB enters cells via receptor-mediated endocytosis (31). In response to vATPase-mediated acidification of endosomes, the DRBD undergoes a conformational change that results in the delivery of both the GTD and CPD into the cytosol (32). The CPD is allosterically activated by host InsP6, resulting in cleavage and release of the amino-terminal GTD (33). The active GTD domain inactivates small GTPases from the Rho family, using UDP-glucose as a substrate (34). The GTD possesses a membrane localization domain (MLD) at the N-terminus of the enzyme, targeting the toxic protein to the cytosolic face of the plasma membrane, where it can encounter membrane-tethered GTPases (35).

To advance our understanding of toxins and generate treatments for toxin-mediated disease, it is important to unravel the ways they interact with host cells. Our knowledge of the suite of receptors engaged by TcdB to bind and enter cells is incomplete. The toxin appears to utilize multiple receptors in interactions dependent on both the toxin strain and the targeted cell type. The Frizzled (FZD) Wnt-binding family of proteins serves as a high-affinity receptor for TcdB (13); the toxin binds the family of receptors through a region spanning residues 1,285–1,804 of the DRBD (36). Chondroitin sulfate proteoglycan 4 (CSPG4) is a TcdB receptor present on myofibroblasts in the subepithelial layer (10) and perivascular pericytes (37). TcdB engages CSPG4 through a dispersed binding interface across residues in the CPD and the hinge (38, 39). A recent study suggested that TcdB engages the internalizing receptor low-density lipoprotein receptor-related protein 1 (LRP1) through its CROP region (40). LRP1 has also been implicated in the cellular uptake of *C. difficile* toxin TcdA (41) and the CROP-less LCT TpeL (42).

Here, we demonstrate the first application of the traditional BioID method to map proximal interactors of protein exotoxins, the LCT TcdB from *C. difficile*, and the model toxin diphtheria toxin using the latest miniTurbo enzyme. We examine the efficacy of this method to resolve the intracellular interacting partners of a toxin in mammalian cells. Additionally, we present a novel approach to mapping cell surface interactions, employing a purified recombinant toxin protein, specifically the receptor-binding region, fused to the TurboID enzyme to identify the host cell receptors hijacked by protein toxins to enter the cell.

## RESULTS

### Mapping the intracellular interactome of the TcdB glucosyltransferase domain using traditional BioID

**Fig 2:**
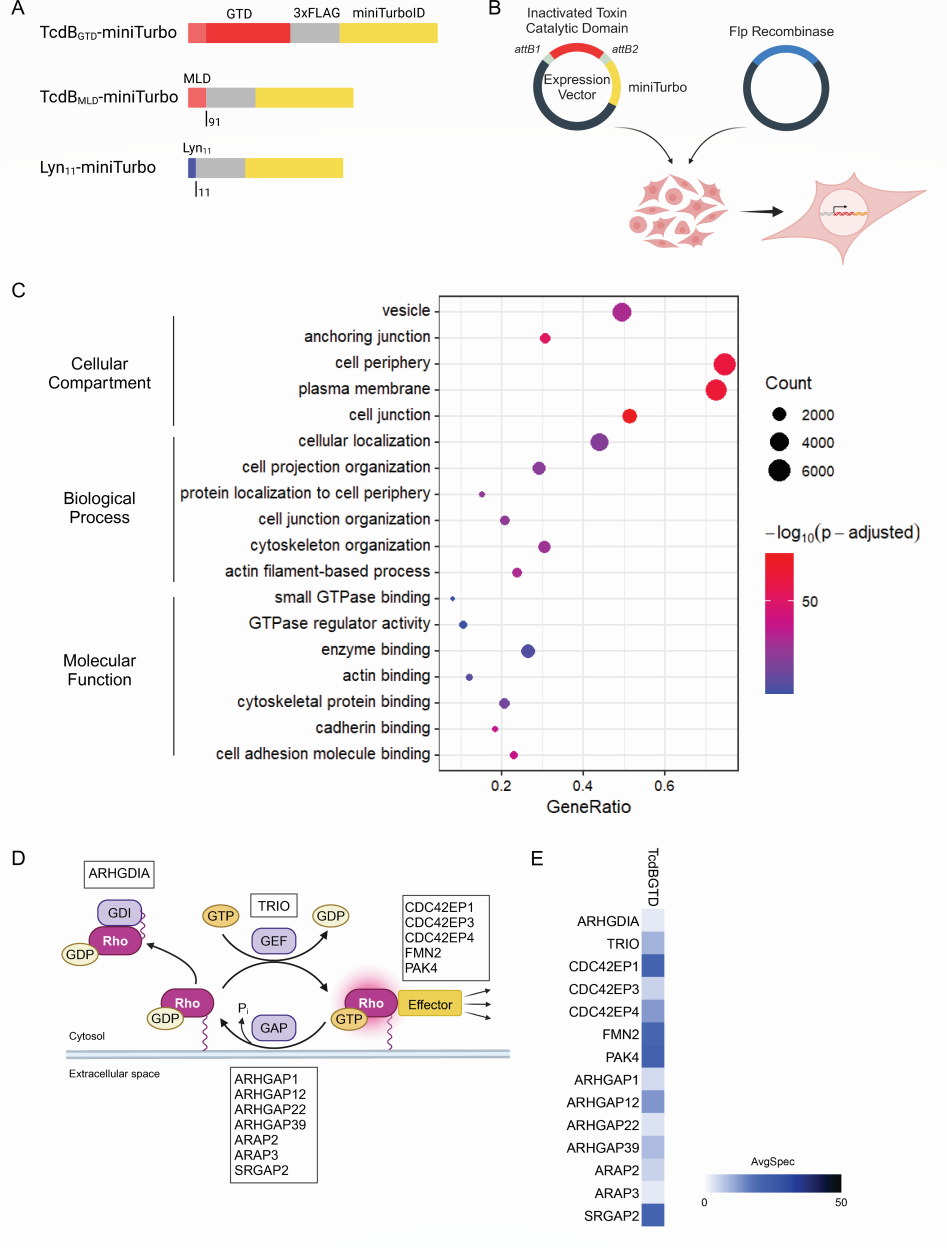
Mapping the interactome of the TcdB GTD with miniTurbo. (**A**) MiniTurbo fusions expressed in HEK293 cells. (**B**) Stable cell line generation with T-REx system, showing an inactivated toxin catalytic domain as the cloned bait protein. (**C**) Enriched molecular functions, biological processes, and cellular compartment localization of TcdB_GTD_-miniTurbo computed from interactors with BFDR ≤ 0.01. (**D**) Illustration of the regulation and signaling of Rho small GTPases at the plasma membrane. Genes for GTPase-activating proteins (GAPs), guanine nucleotide exchange factor (GEFs), GDP dissociation inhibitors (GDIs), and effector proteins detected as significant prey of TcdB_GTD_-miniTurbo are listed in the bordered boxes. (**E**) Average spectral count of regulatory proteins and effectors identified as high-confidence interactor by TcdB_GTD_-miniTurbo, BFDR ≤ 0.01.

TcdB contains multiple domains that play key roles in receptor binding (“delivery and receptor-binding domain”), cleave region to release active domain (“cysteine protease domain”), and enzymatically active domain (“glucosyltransferase domain”; Fig. 1). Initially, we set out to use the canonical BioID approach to study the interactions of the cytotoxic GTD with host factors in the cytosol. Given the profound cytotoxicity of the GTD, which would result in rapid cellular death upon induction (or likely even before induction), we created a chimeric construct of a nontoxic GTD mutant (D286N/D288N) (43) fused to miniTurbo (Fig. 2A). This construct was integrated into the Flp-In locus of the HEK293 cells by the T-REx system where the miniTurbo fusion protein can be stably and inducibly expressed (Fig. 2B). After overnight induction of TcdB_GTD_-miniTurbo bait expression, biotin labeling was performed for 1 hour to label proteins proximal to TcdB_GTD_-miniTurbo. Biotinylated proteins were enriched and identified using liquid chromatography coupled with mass spectrometry (see Materials and Methods). Significance Analysis of INTeractomes, or SAINT, scoring assessed which proteins detected by mass spectrometry (MS) were significantly higher than the non-specific labeling pattern of a miniTurbo alone control (44). We identified 311 high-confidence interactors at ≤0.01 Bayesian false discovery rate (BFDR) threshold (Table S1). To explore the pathways and the cellular biology associated with the high-confidence interacting proteins, we analyzed the high-confidence prey list using gene ontology (GO) term enrichment.

GO analysis indicated that the GTD localizes to the plasma membrane, cytoskeleton, cell junctions, and vesicles, which include proteins known to localize to endosomes (Fig. 2C). The biological processes associated with the GTD include actin cytoskeletal organization, cell-cell adhesion, cell morphogenesis, and regulation of GTPase activity. The enriched molecular functions indicate that the TcdB GTD is implicated in various functions at the cell periphery, such as actin-binding, cadherin-binding, phospholipid-binding, and cell adhesion molecule binding (Fig. 2C). The related molecular functions of small GTPase binding, GTPase regulator activity, and GTPase activator activity were also enriched. GO analysis of cellular reactions showed enrichment for the Rho GTPase cycle. Specific GTPase cycles were resolved, including cycles for Cdc42, Rac1-3, and 10 Rho proteins. Looking closer at which proteins were differentially biotinylated, we noted that TcdB_GTD_-miniTurbo labeled several GAPs, GEFs, GDIs, and small GTPase effector proteins (Fig. 2D and E); however, only one small GTPase itself, RhoA, was identified. Therefore, the enrichment of the Cdc42 GTPase cycle, for example, is due to the identification of Cdc42 regulatory proteins and effectors, not the small GTPase itself.

**Fig 3:**
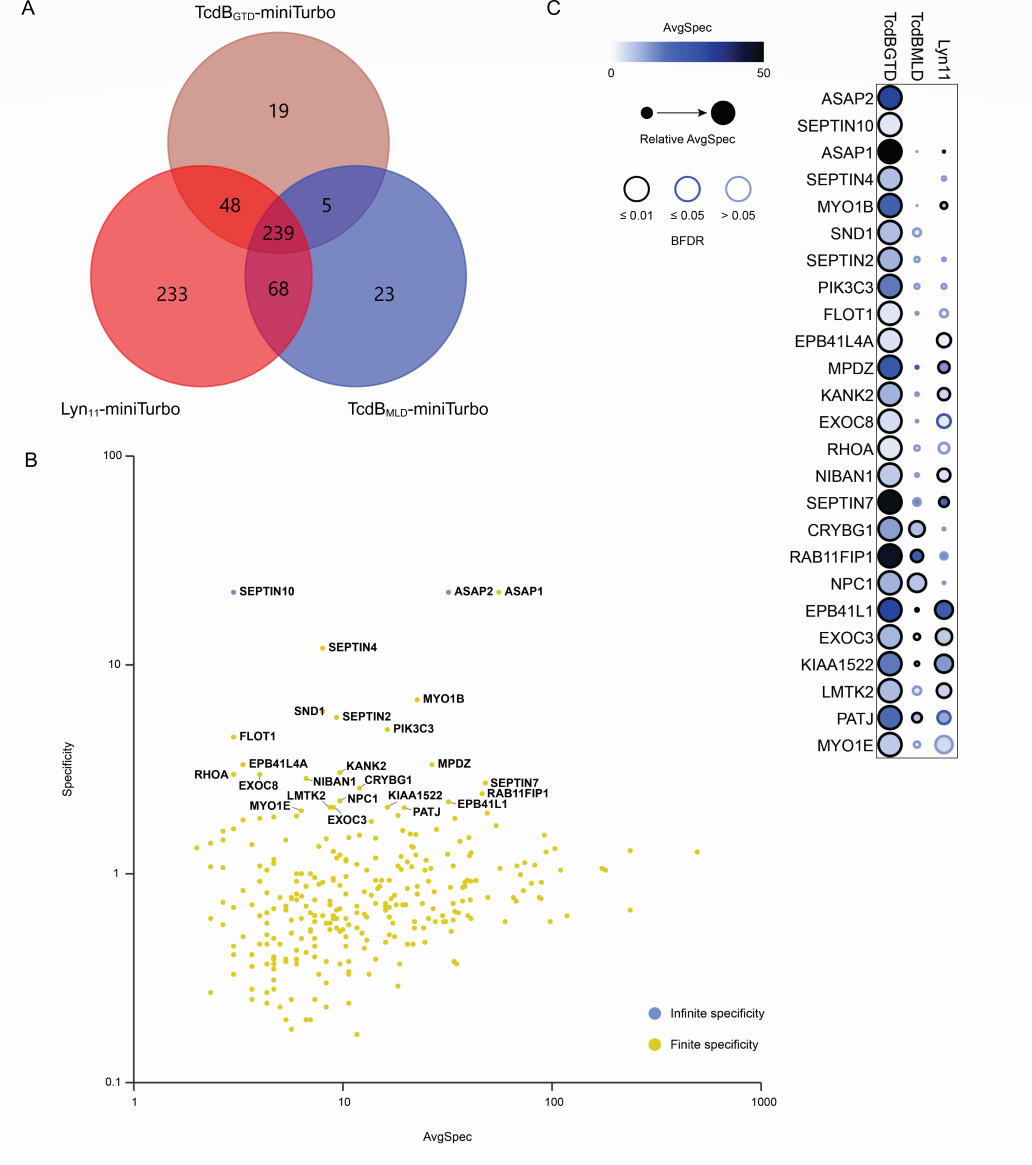
Prey specificity for TcdB_GTD_. (**A**) Venn diagram illustrating the number of genes with BFDR ≤ 0.01 for each bait protein and their associated overlap. (**B**) Visualization of TcdB_GTD_-miniTurbo prey specificity using their abundance information detected in plasma membrane-targeted TcdB_MLD_-miniTurbo and Lyn_11_-miniTurbo BioID experiments. Prey proteins with a specificity score ≥2 are labeled. Preys with infinite specificity, indicating that they were solely detected in TcdB_GTD_-miniTurbo are in blue. (**C**) Dot plot showing the prey proteins detected with relatively higher abundance by TcdB_GTD_-miniTurbo BioID, compared to TcdB_MLD_-miniTurbo and Lyn_11_-miniTurbo BioID. Prey proteins with a specificity score ≥2 are shown in descending order. Relative average spectral count, represented by the size of the dot, is the abundance of a prey protein for one bait compared to its average abundance across all baits.

Next, we asked the extent to which the GTD interactome was due to being juxtaposed proximal to the membrane by its N-terminal 91-residue MLD. To this end, we generated a stable cell line expressing the MLD of TcdB, TcdB_1–91_ fused to miniTurbo (i.e., TcdB_MLD_-miniTurbo; Fig. 2A). As expected, there was an overlapping subset of proteins and processes identified with the membrane-localized MLD alone with the full-length GTD (Fig. 3A; Table S1). Similar to TcdB_GTD_-miniTurbo, TcdB_MLD_-miniTurbo showed significant enrichment of small GTPase binding, GTPase regulator activity, and GTPase activator activity, as well as enrichment of the Rho GTPase cycle.

Next, to further distinguish whether the MLD was specifically interacting with the identified proteins in the membrane vs labeling these targets as a consequence of being concentrated in the two-dimensional inner leaflet of the plasma membrane, we selected a plasma membrane localization sequence composed of the first 11 residues of the peripheral membrane protein Lyn (45, 46). This sequence includes myristoylation and palmitoylation sites, targeting the peptide to the plasma membrane. The resulting construct, Lyn_11_-miniTurbo, produced an interactome with a subset of targets overlapping with TcdB_GTD_-miniTurbo and TcdB_MLD_-miniTurbo (Fig. 3A). While the number of high-confidence interacting proteins detected by Lyn_11_ as bait was approximately double that of the GTD or MLD, GO analysis of Lyn_11_-miniTurbo interactors showed enrichment of certain molecular functions, biological processes, and cellular reactions as TcdB_GTD_-miniTurbo and TcdRB_MLD_-miniTurbo, suggesting that plasma membrane targeting on its own is sufficient to proximally associate with proteins involved in small GTPase cycling.

Based on these findings, we focused on high-confidence preys with high specificity for TcdB_GTD_-miniTurbo, i.e., interactors unique to TcdB_GTD_ or preys more highly enriched by this bait than by the MLD or Lyn_11_ constructs. To determine a proximal interactor identified with high specificity in TcdB_GTD_-miniTurbo BioID, we utilized the “specificity” score that calculates the abundance of prey in TcdB_GTD_-miniTurbo BioID normalized to the average abundance of the prey identified in other BioID conditions (Lyn_11_-miniTurbo BioID and TcdB_MLD_-miniTurbo BioID) (47). The specificity and average spectral count for TcdB_GTD_-miniTurbo prey proteins are presented in Fig. 3B using ProHits-viz tool. Infinite specificity indicates that the prey protein was only detected by TcdB_GTD_-miniTurbo. High-confidence interactors with a specificity score ≥2 for TcdB_GTD_-miniTurbo BioID are shown as a dot plot in Fig. 3C. RhoA, one of the small GTPases that the toxin inactivates through glucosylation (48), was identified with high specificity in TcdB_GTD_-miniTurbo BioID, suggesting that the GTD domain enables substrate targeting once localized to the plasma membrane compartment by the MLD. The TcdB GTD also specifically captured ARAP3 (not shown), a GTPase-activating protein that acts on RAC1, RHOA, and CDC42 (49). Scaffolding proteins such as septins and flotillin and proteins involved in endosomal trafficking, such as RAB11FIP1 and PIK3C3, were identified with high specificity in TcdB_GTD_-miniTurbo screen. These data showcase proximal interactors specifically enriched in TcdB_GTD_-miniTurbo BioID relative to the minimal plasma membrane-targeted BioID (Lyn_11_-miniTurbo and TcdB_MLD_-miniTurbo), providing potential host targets of TcdB.

### Mapping the cell-surface interactome of toxins using recombinant protein BioID

An important first step in the intoxication pathway of TcdB and many other bacterial toxins is binding to a cell-surface receptor (Fig. 1). Elucidating the cell-surface receptors of toxins is an area of major focus in toxin pathogenesis as these molecular interactions are often the most highly coveted targets for therapeutic intervention. To determine if proximity labeling using TurboID could be used to identify which proteins toxins interact with on the cell surface, we designed a series of fusion constructs with the receptor-binding moieties of TcdB and DT—the archetype A-B toxin. However, to identify the receptors of toxins, we recognized that modifications to the canonical BioID approach of expressing fusions in cells were needed to mimic the way in which toxins bind target cells exogenously. To this end, instead of expressing the protein of interest in host cells, we generated recombinant proteins of toxin receptor fragments fused to TurboID by protein expression in *E. coli* and purification using affinity chromatography. The resulting chimeras were then added exogenously to target cells so that they could bind and subsequently label the host cell receptors.

**Fig 4:**
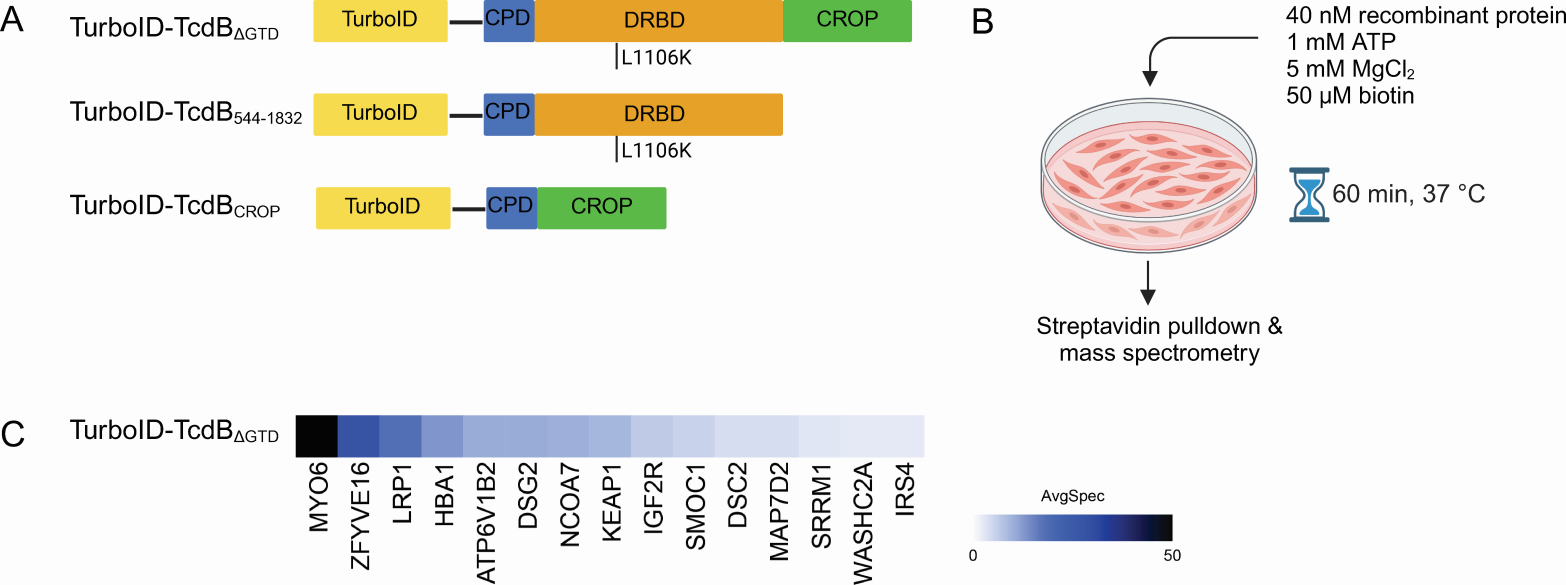
Cell surface labeling with TurboID fusion proteins to a protein toxin. (**A**) Recombinant TurboID fusions to fragments of TcdB, purified from *E. coli*. (**B**) Cell surface labeling with TurboID. Recombinant protein was incubated on confluent cells with supplemental ATP, MgCl_2_, and biotin for an hour at 37°C. (**C**) Average spectral count for prey proteins with BFDR ≤ 0.01 of bait TurboID-TcdB_ΔGTD_ on HEK293T cells.

Initially, we cloned a fusion of TurboID in place of the GTD on TcdB (i.e., TcdB_534–2,366_ or TurboID-TcdB_ΔGTD_) containing the L1106K point mutation to prevent translocation and release of TurboID into the cytoplasm (Fig. 4A). This preserved the CPD, DRBD, and CROP region, as all three domains have been implicated in receptor binding. We incubated TurboID-TcdB_ΔGTD_ on HEK293T cells, with supplemental ATP and MgCl_2_ to facilitate the biotinylation reaction (Fig. 4B). Recombinant TurboID alone was added to cells in the same manner and served as the control condition. SAINT scoring yielded a short list of proteins with BFDR ≤ 0.01 (Fig. 4C; Table S2). Notably, in this short list were a series of targets associated with endosomes and endosomal trafficking, including ZFYVE16 and ATP6V1B2 and the cell-surface protein LRP1, a membrane protein recently suggested to be a TcdB receptor.

Noting research by Guo et al. suggesting that LRP1 engages TcdB through the toxin’s CROP domain, we then designed TurboID fusions with just the CROP domain, TurboID-TcdB_CROP_, and a fusion that excluded the CROP domain, TurboID-TcdB_544–1,832_. We tested these two constructs, in addition to TurboID-TcdB_ΔGTD_, on 18Co cells, a healthy colonic cell line that expresses FZD and CSPG4 (Table 1; Table S3). The variable spectral counts for LRP1 produced by these chimeras and the TurboID alone control resulted in non-significant SAINT scores and BFDR scores below the cut-off. Although not identified as a high-confidence interactor using the stringent BFDR < 0.01 threshold, LRP1 was identified with the highest abundance in TurboID-TcdB_CROP_. Interestingly, the only protein with BFDR ≤ 0.01 for TurboID-TcdB_CROP_ and TurboID-TcdB_544–1,832_ was tissue-type plasminogen activator, or tPA, a secreted protein that interacts with LRP1 (50). Finally, the CROP-less bait TurboID-TcdB_544–1,832_ identified FZD2, a known receptor for TcdB, as a high-confidence interacting protein with BFDR ≤ 0.05 threshold.

**TABLE 1:** SAINT scoring for prey protein LRP1 labeled on 18Co cells by the recombinant bait proteins TurboID-TcdB_ΔGTD_, TcdB_CROP_, and TurboID-TcdB_544–1,832_

Bait	No. reps	Spec count	Ctrl count	Saint score	BFDR
TurboID-TcdB_ΔGTD_	3	8|1|23	1|1|32	0	0.89
TurboID-TcdB_CROP_	3	2|107|162	1|1|32	0.64	0.24
TurboID-TcdB_544–1,832_	3	0|2|66	1|1|32	0.26	0.65

**Fig 5:**
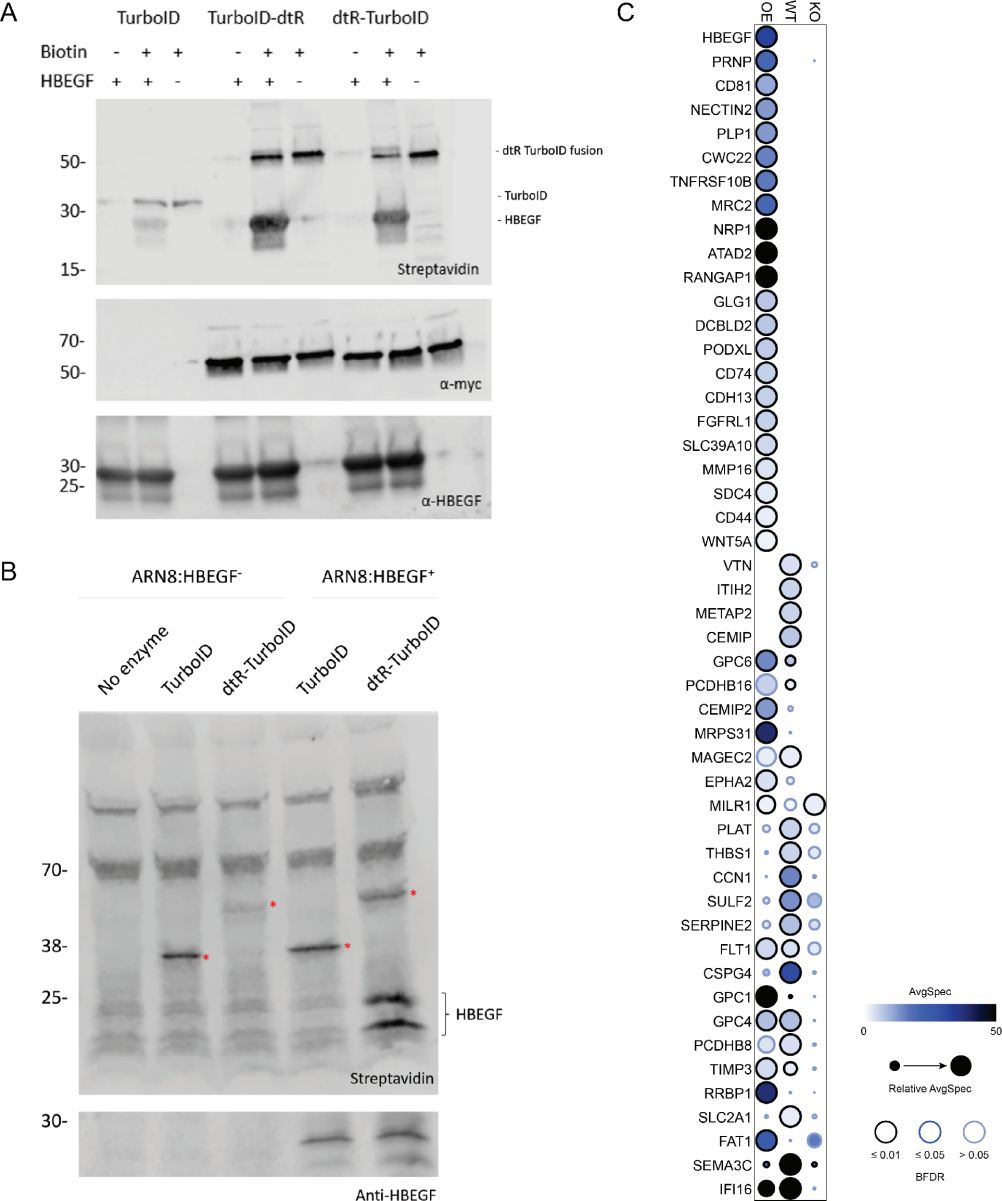
dtR-TurboID fusion labels DT receptor HBEGF. (**A**) Recombinant N- and C-terminal fusions of dtR and TurboID biotinylate soluble HBEGF. *In vitro* assay of 300 nM enzyme with and without 50 µM biotin and 20 µM HBEGF was analyzed by western blotting to detect biotinylation signal. TurboID-toxin chimeras were expressed with a C-terminal myc tag. (**B**) dtR-TurboID labels HBEGF on ARN8:HBEGF^+^ cells. One hundred fifty nanomolar of recombinant TurboID or dtR-TurboID was incubated with suspended HBEGF KO (ARN8:HBEGF^-^) or HBEGF-expressing (ARN8:HBEGF^+^) ARN8 cells with 50 µM biotin for 10 minutes at room temperature (RT). Self-biotinylation is indicated with an asterisk. (**C**) High-confidence prey proteins identified with BFDR ≤ 0.01 threshold performed on three ARN8 cell lines by dtR-TurboID: HBEGF-overexpressing (OE), wild-type (WT), and HBEGF KO (KO). Data shown are from two replicates on OE and KO cells and a single replicate on WT cells.

To determine whether our novel method could be used on other toxins, we applied this approach to the archetype toxin DT. DT uses a single-cell surface receptor, HBEGF, to bind host cells and internalize. We showed that a fusion of the receptor-binding domain of DT N-terminal to TurboID biotinylated recombinant HBEGF when incubated together *in vitro* (Fig. 5A) and labeled HBEGF in cell lysate of ARN8 cells engineered to overexpress the receptor, abbreviated ARN8:HBEGF^+^ (Fig. 5B). Furthermore, we captured HBEGF as a high-confidence interacting protein in a cell surface BioID screen using recombinant TurboID-DT toxin on ARN8:HBEGF^+^ cells, though the DT receptor was not detected on WT cells (Fig. 5C; Table S4). Other prey proteins include the cell surface receptors CD44 and CD81. CD44 interacts with numerous extracellular matrix proteins through its ectodomain, and CD81 acts as a viral receptor.

## DISCUSSION

Proximity-dependent biotinylation techniques, or BioID and APEX, have emerged as powerful methods and techniques for studying the organization of the proteome (46) and the proteomes of subcellular compartments (22, 51–54). Its use in mapping the interactions of “foreign” proteins with the host as is the case with toxins has been limited to studies on type III secretion system bacterial effectors (27, 55). To our knowledge, the work presented here represents the first application of this method to exotoxins that mediate their own entry into cells. Different from extracellular BioID relying on host expression of transmembrane protein (56), we explored the use of recombinant proteins for extracellular proximity interactions between exotoxin and host cell receptor, providing the first application of BioID with free recombinant protein. We investigated the potential of this technique to resolve host-toxin interactions, including the cell surface receptors hijacked by toxins to enter cells, focusing on the large clostridial toxin TcdB and the model toxin diphtheria toxin.

To obtain a “toxin’s-eye” view of the cell and map interactions between the toxin’s catalytic domain and host intracellular proteins, we expressed a fusion of the TcdB GTD and miniTurbo. TcdB possesses an MLD that targets the toxin to the inner plasma membrane, bringing the GTD in proximity with its substrate, membrane-tethered small GTPases. Proximity labeling resolved the known localization of the GTD to the plasma membrane, cell junctions, cytoskeleton, and endosomes. GO analysis of the high confidence interacting proteins aligned with the known action of the GTD on small GTPases, with the enriched molecular functions of small GTPase binding, GTPase regulator activity, and GTPase activator activity, as well as enrichment of the Rho GTPase cycle.

High-confidence proximal interactors included GAPs, GEFs, GDIs, and effectors, the network of proteins involved in the regulation and signal transmission of the small GTPase molecular switches. While RhoA itself was captured, other small GTPase targets of the GTD were not biotinylated. It is plausible that miniTurbo’s access to the small GTPase targets was obstructed by the complex of GTPase regulatory proteins assembled at the plasma membrane and acting on the GTPase. We can also speculate that the GTPases themselves were not identified due to their short residency time within the activated biotin cloud generated by the TcdB_GTD_-miniTurbo fusion, as GTDs are extremely processive enzymes and thus would be expected to have a rapid off-rate from its targets, which can be remedied by longer biotinylation reactions to accumulate transient interactions. The TcdB_GTD_-miniTurbo fusion featured a catalytically inactive GTD, due to the nature of the experiment, and it is not known if or to what extent this would impact the binding affinity between the GTD enzyme and its GTPase target. Alternatively, small GTPases may lack accessible lysine residues required for biotinylation. Lobingier et al. encountered a similar phenomenon with their research on G-protein-coupled receptor (GPCR) interacting proteins, wherein heterotrimeric G proteins were not scored as high-confidence interactors by SAINT (45). They hypothesized that this was due to the sub-second kinetics of the initial binding event between the GPCR and the G protein.

We employed the MLD alone and the plasma membrane-targeted peptide Lyn_11_ as baits to assess the value of plasma membrane-localized miniTurbo controls in refining the interactome of the TcdB GTD. Notably, we found that there was significant overlap in the prey proteins between all three bait and that, consequently, the biological processes, molecular functions, and cellular reactions resolved from TcdB_MLD_-miniTurbo or Lyn_11_-miniTurbo resembled those of the full-length GTD. These findings serve as a note of caution when evaluating the specificity of interactions detected by plasma membrane-localized bait with a cytosolic miniTurbo control and the need to implement compartment-specific BioID to compare to. Furthermore, it is possible that the overexpression of the engineered bait proteins could overwhelm the signal for specific bait-prey interactions. Using a range of tetracycline concentrations for induction to titrate bait expression levels may be a fruitful approach to address this in future research.

We focused on the interactors with specificity for the TcdB GTD, i.e., prey unique to this bait or more highly enriched by the GTD compared to the MLD or Lyn_11_. GTD-specific prey included a small GTPase, GTPase regulatory proteins, and numerous structural components of the plasma membrane. TcdB_GTD_-miniTurbo labeled the small GTPase RhoA. Through signaling its downstream effectors, RhoA modulates the actin cytoskeleton, influencing cell-cell adhesion, cell motility, and cell polarity (57, 58). TcdB glucosylation of RhoA therefore disrupts cell structure and adhesion (59, 60). TcdB_GTD_-miniTurbo also captured a RhoA GTPase activating protein, ARAP3. Thus, with stringent prey refinement, we have successfully captured a known GTPase target and a proximal regulatory protein of the exotoxin TcdB with TurboID.

Structural components of the plasma membrane were uniquely enriched with the TcdB GTD, including septins 2, 4, 7, and 10. Septins are GTP-binding proteins that form hetero-oligomeric complexes and provide scaffolding on the inner plasma membrane. They interact with F-actin and thus co-localize with actin-modulating proteins such as the small GTPase Cdc42 (61). Nölke et al. showed that the toxicity of another *C. difficile* toxin, *C. difficile* transferase, is septin dependent (62). Our identification of several septins as high-confidence interactors of the TcdB GTD in this TurboID screen suggests that these structural proteins could also play a role in the toxic mechanism of *C. difficile* toxin B. GTD specific preys including numerous proteins involved in membrane trafficking and endocytosis, including Rab11 family-interacting protein 1, flotillin 1, unconventional myosin 1e, and Phosphatidylinositol 3-kinase catalytic subunit type 3. Finally, PFDN2 or Prefoldin subunit 2 was captured by TcdB_GTD_-miniTurbo. PFDN2 transfers nonnative proteins to cytosolic chaperonin (63), suggesting that the GTD receives help to refold after delivery to the cytosol.

BioID fusion constructs have historically been enclosed by or tethered to the cell, with fusion proteins expressed from exogenously introduced plasmids or CRISPR-edited genomic regions (64, 65). Most applications of this technology investigate intracellular compartments. A handful of studies have explored protein-protein interactions with TurboID-linked plasma membrane proteins, wherein the biotin ligase extends into the extracellular environment, demonstrating that the enzyme is functional and achieves sufficient labeling on the cell surface (56, 66–68). An unexplored application of TurboID is identifying cell surface receptors of extracellular proteins by engineering and expressing recombinant TurboID-ligand fusions and applying them to cultured cells. Previously, the only use of recombinant BioID fusions for receptor labeling was a 2022 proof of concept study with EGFR and Her2 that stopped short of MS analysis (69).

We first employed this novel application of TurboID by incubating the fusion protein TurboID-TcdB_ΔGTD_ on mammalian cells. We predicted that this construct would label the known TcdB receptors FZD and CSPG4 and capture other information about the toxin’s interaction with the cell surface. Interestingly, neither FZD nor CSPG4 was a high-confidence interacting protein. One internalizing receptor that stood out in the data sets was LRP1. Low-density lipoprotein receptor-related protein 1, or LRP1, is a 600 kDa internalizing and signaling receptor, widely expressed in different cell types (70, 71). Almost a decade after LRP1 was deemed a receptor for *Pseudomonas aeruginosa* exotoxin A, the internalizing receptor was also found to bind the CROP-less LCT TpeL through a haploid genetic screen (42). There is conflicting evidence surrounding the role of LRP1 as a cellular receptor for the *C. difficile* toxins TcdA and TcdB and the involvement of the CROP in this potential interaction. A 2020 study found that TcdA, but not TcdB, utilizes LRP1 as an internalizing receptor (41). In contrast, a recent study by Guo et al. suggested that TcdB utilizes LRP1 to intoxicate cells and binds the receptor through its CROP region (40).

We engineered TurboID fusions with only the CROP domain, TurboID-TcdB_CROP_, or excluding the CROP domain, TurboID-TcdB_544–1,832_. LRP1 was detected with spectral counts >100 for two of three replicates by TurboID-TcdB_CROP_. While high variance in sample and control spectral counts resulted in a non-significant SAINT score (0.64) and BFDR (0.24), LRP1 abundance was the highest in TurboID-TcdB_CROP_ compared to BioID profiling of other truncation constructs. Overall, cell surface labeling by recombinant fusions yields low spectral counts across prey—single digit counts as opposed to several hundred peptides/sample as seen in traditional intracellular biotinylation experiments. As quantitative proteomics with low spectral count proteins is challenging due to the high variability, it is difficult to confidently identify biotinylation levels above background.

The CROP-less bait TurboID-TcdB_544–1,832_ captured FZD2, a known receptor for TcdB (BFDR 0.02). Recent studies exploring the role of CROP dynamics in TcdB binding to CSPG4 and FZD suggest that the CROP domain, which appears to be in equilibrium between an open and closed conformation, can affect receptor binding; in the “closed” conformation, the CROP domain occludes these sites (72, 73). Consistent with this, we hypothesize that the CROP-less TurboID-TcdB_544–1,832_ allows for greater access to the FZD-binding site. Conversely, neither FZD nor CSPG4 appeared as high-confidence interacting proteins of TurboID-TcdB_ΔGTD_. We speculate that FZD and CSPG4 were not identified due to spatial constraints in TurboID-TcdB_ΔGTD_ BioID. TcdB_ΔGTD_ is a large, 207 kDa fragment, so it is reasonable to assume that accessible, surface-exposed lysine residues on FZD and CSPG4 may not be positioned within a 10 nm cloud of the TurboID enzyme when TurboID-TcdB_ΔGTD_ is bound to these receptors. The issue of ligand size for BioID/TurboID proximity labeling was also raised by Alwash and Gariépy, who postulated this was the reason they were unable to move from affibodies to antibodies in their receptor labeling proof-of-concept experiments (69). If LRP1 indeed binds to the CROP, it could be easier for this receptor to be labeled by TurboID because of the flexibility of the CROP region extending from the rest of the toxin, allowing TurboID to be in proximity to the CROP-LRP1-binding site. It is also possible that the large, extracellular chain of LRP1 makes first contact with TcdB, anchoring it to the cell surface, and in subsequently internalizing the toxin, bypasses FZD and/or CSPG4 on the timescale of TurboID.

We extended this new application of TurboID for receptor labeling to the model toxin DT, which is known to bind HBEGF on the cell surface. *In vitro* experiments with soluble HBEGF and with cell lysate of ARN8 HBEGF-overexpressing cells were extremely promising, showing clear biotinylation of HBEGF on western blots. This translated to the identification of HBEGF as a high-confidence interacting protein using mass spectrometry and statistical analysis compared to negative controls..

### Conclusions

TurboID is commonly leveraged to map the interactome of endogenous mammalian proteins. Here, we extended this technique to foreign bacterial toxins to capture their interactions with the cell surface and the host factors they engage with intracellularly. Proximity labeling with the GTD of *C. difficile* toxin B resolved the known localization of the toxin to the inner plasma membrane. We explored the use of membrane-localized controls to refine the GTD interactome, yielding a short list of GTD-specific interactors. Prey included RhoA, a host cell target of TcdB, and four septins.

We have presented the first application of free, recombinant TurboID for cell surface labeling, focusing on protein toxins. Fusions of TurboID with C-terminal fragments of TcdB provided support for the role of LRP1 as a receptor. Furthermore, we showed that the receptor-binding domain of the model toxin diphtheria toxin fused to TurboID biotinylated its singular known receptor, HBEGF.

## MATERIALS AND METHODS

### Cloning of recombinant proteins

DT plasmid carrying the E148S mutation was a kind gift from Dr. R. John Collier (Harvard Medical School, Boston, MA). Point mutations were made previously in the Melnyk lab to generate WT-DT, using QuikChange Lightning Multi Mutagenesis Kit (Agilent Technologies). TcdB strain VPI10463 was previously cloned from an *E. coli* codon-optimized plasmid (GenScript) into a pET28 vector. *E. coli* expression optimized TurboID (Addgene Catalogue #107177) from the lab of Alice Ting was cloned into a Champion pET-SUMO vector (Invitrogen) alone or as fusions with DT or TcdB using the In-Fusion HD Cloning Kit (Clontech).

### Gateway cloning

Expression plasmids for Flp-In cell line generation were generated using the Gateway Cloning method (Invitrogen). Dr. Ji-Young Youn (SickKids, Toronto, ON) gifted the pDONR223 plasmid (Invitrogen) and a pcDNA5 pDEST plasmid encoding 3xF-miniTurbo with an N-terminal recombination site. A K51E E148K double-point mutant was made in the WT DT plasmid using the QuikChange Lightning Multi Mutagenesis Kit (Agilent). A D286N D288N double mutant was made in the WT TcdB plasmid using the QuikChange Lightning Kit (Agilent). Inactive mutant catalytic domains of DT or TcdB were amplified with attB primers using PCR. BP and LR reactions were performed as per the manufacturer’s instructions (Invitrogen).

### Expression and purification of chimeras

Various TurboID-toxin fusions and TurboID alone were expressed as N-terminal His_6_-SUMO fusions in *E. coli* BL21 (DE3) cells and induced with 0.1 mM isopropyl-β-D-1-thiogalactopyranoside (IPTG) overnight at 18°C. TurboID-toxin chimeras were also expressed with a C-terminal myc tag. Cells were harvested by centrifugation and resuspended with lysis buffer (20 mM Tris-HCl buffer [pH 7.5], 150 mM NaCl) then lysed by sonication using the Qsonica Q500. Proteins were purified first by His-tag affinity chromatography using a His-Trap FF column (Cytiva/GE Healthcare) and eluted with an imidazole gradient. The SUMO tag was cleaved overnight at 4°C by adding 1 U SUMO Protease (Life Sensor). Proteins were secondarily purified by either reverse affinity chromatography with the His-Trap column or by ion exchange chromatography with a HiTrap HP column (Cytiva).

### *In vitro* biotinylation

Three hundred nanomolar purified recombinant TurboID or dtR-TurboID, 30 µM HBEGF, 2 mM MgCl_2_, 2 mM ATP, and 50 µM biotin were incubated in HEPES buffer at 37°C for 30 minutes.

For biotinylation of cell lysate, ARN8 cells were harvested from a T175 culture flask with Non-Enzymatic Cell Dissociation Buffer (Sigma) and aliquoted to 2.5–3 million cells/tube. Each cell pellet was resuspended in 100 µL 1× radioimmunoprecipitation assay (RIPA) buffer (abcam) and incubated for 30 minutes at 4°C. Cell supernatant was incubated with 150 nM recombinant protein and 50 µM biotin for 10 minutes at RT.

### Western blotting

Samples were resuspended in Laemmli sample buffer with 2-mercaptoethanol (Bio-Rad), boiled at 92°C for 5–10 min, and run on a precast polyacrylamide gel. Protein was transferred onto a nitrocellulose membrane using a semi-dry transfer method. Western blot analysis was performed using the following antibodies: IRDye 800CW Streptavidin diluted 1:5,000 (LI-COR); anti-myc mouse IgG diluted 1:2,000 (Millipore), and IRDye 680RD goat anti-mouse secondary antibody diluted 1:20,000 (LI-COR); anti-HB-EGF goat IgG diluted 1:1,000 (R&D Systems) and rabbit anti-goat horseradish peroxidase (HRP) secondary antibody diluted 1:100,000 (Bio-Rad). Western blots were imaged on the LI-COR Odyssey FC. If probed with HRP-conjugated antibodies, the blot was then developed using the SuperSignal West Femto kit (ThermoScientific) and imaged again.

### Cell-surface proximity labeling with TurboID fusions

For DT experiments, ARN8 cells were grown to confluence in a 15 cm dish. One hundred fifty nanomolar recombinant protein, 5 mM MgCl_2_, 1 mM ATP, and 50 µM biotin were added, and cells were incubated for 10 minutes at 37°C. For TcdB experiments, HEK293 or 18Co cells were grown to confluence in a 15 cm dish. Fifty nanomolar recombinant protein, 5 mM MgCl_2_, 1 mM ATP, and 50 µM biotin were added, and cells were incubated for 60 minutes at 37°C.

After labeling, dishes were placed on ice. Cells were rinsed with phosphate-buffered saline (PBS) then the cells were collected in 1 mL PBS by scraping. The cell pellet was collected by centrifugation and frozen on dry ice.

### Flp-In stable cell line generation and proximity labeling

HEK293 Flp-In T-REx cells (Invitrogen) were generously gifted by Dr. Ji-Young Youn. Cells were grown at 37°C in Dulbecco's modified Eagle medium (DMEM) or Eagle's minimum essential medium (EMEM) supplemented with 10% fetal bovine serum (FBS) and 1% penicillin/streptomycin. To generate stable cells lines, cells were seeded at 200,000 cells/well in a six-well plate in grown media lacking antibiotics. The next day, cells were transfected with 1 µg pOG44 (Invitrogen) and 100 ng of the expression clone using jetPRIME transfection reagent (Polyplus). On day 2, transfected cells were passaged to 10 cm plates in complete media. On day 3, media was replaced with complete media supplemented with 200 µg/mL Hygromycin (Multicell). The duration of selection was 1–2 weeks, after which time visible colonies were pooled and cells passaged in complete media. Dr. Ji-Young Youn also generously provided HEK293 cells expressing miniTurbo-3xF that were generated with the above method.

For proximity-dependent biotinylation experiments, cells were grown to 60%–80% confluence in 10 cm dishes, and the expression of miniTurbo proteins was induced with 1 µg/mL tetracycline. Twenty-four hours later, media was supplemented with 50 µM biotin. After 10–60 minutes, the media was aspirated, cells were washed with PBS, and cells were harvested by trypsinization. The cell pellet was collected by centrifugation, rinsed with PBS, pelleted again, and frozen on dry ice.

### Biotin-streptavidin affinity purification for mass spectrometry

Biotinylated proteins were purified following procedures published in the Journal of Proteome Research (74). Cell pellets were lysed in modified RIPA buffer (50 mM Tris-HCl [pH 7.5], 150 mM NaCl, 1 mM EGTA, 1.5 mM MgCl_2_, 1% NP-40, and 0.1% sodium deoxycholate) with Sigma-Aldrich protease inhibitors (P8340 1:500) at 1:10 (pellet weight in mg: lysis buffer volume in µL). Cells were sonicated for 30 s (cycles of 10 s on and 5 s rest on ice) at 30% amplitude. Two hundred fifty units (1 µL/sample) of Benzonase (Millipore Sigma) was added to each sample. After a 15-minute incubation at 4°C, SDS concentration was increased to 0.4%. Lysate volume was normalized to the lowest sample volume then lysates were centrifuged for 20 minutes at 20,810 × *g* at 4°C. The supernatant was transferred to a 50% slurry of streptavidin beads (Cytiva), pre-washed in the modified RIPA buffer, and incubated with streptavidin beads rotating for 3 hours at 4°C. The beads were pelleted (400 × *g* for 1 min), and the supernatant was removed. The beads were transferred to a new microcentrifuge tube with 1 mL modified RIPA buffer, then washed once with SDS wash buffer (50 mM Tris-HCl [pH 7.5], 2% SDS), twice with modified RIPA buffer, and three times with 50 mM ammonium bicarbonate buffer. After the final wash, all residual buffer was removed, and the proteins were digested on beads with 1 µg trypsin (Sigma Aldrich T6567) dissolved in 50 mM ammonium bicarbonate buffer at 37°C overnight.

The following day, 0.5 µg trypsin was added to the samples. After 3–4 hours of incubation, the beads were pelleted, and the supernatant containing peptides was transferred to a new tube. Beads were rinsed twice with high performance liquid chromatography (HPLC) grade H_2_O. Supernatant and washes were pooled, and then samples were acidified to 2% formic acid and dried by vacuum centrifugation without heat.

### Mass spectrometry

Peptides from the proximity-dependent biotinylation samples were analyzed with an Orbitrap Exploris 480 (Thermo Fisher Scientific) operating in data-dependent acquisition mode as described in Schreiber et al. (74). Digested peptides were resuspended in 12 µL of 5% formic acid, and 2–6 µL of this solution was further diluted with 5% formic acid to a final volume of 6 µL. Using an Easy-nLC 1,200 liquid chromatography system, 5 µL of each sample was loaded onto a 10.5 cm 75 µm ID emitter tip column packed in-house with 3 µm ReproSil Gold 120 C18 (Dr. Maisch HPLC GmbH). Peptides were eluted at 200 nL/min over a 120 min gradient starting at 2.4% acetonitrile in 0.1% formic acid and increasing to 35% acetonitrile. The acetonitrile concentration was then increased to 80% over an 8 min gradient and maintained at this concentration for 16 min at 200 nL/min, for a total run time of 144 min. The mass spectrometer was configured with a positive polarity ion voltage of 2.4 kV, 400–1,500 Da MS1 full scan range at 120,000 m/z resolution, normalized automatic gain control target of 125%, precursor selection of ions with charge 2+ to 5+ that exceed an intensity threshold of 1.0 × 10^3^, 9 s dynamic exclusion after one occurrence, 3 s cycle time between MS1 scans, and 33% collision energy.

### Mass spectrometry data analysis

Raw MS data were analyzed with MSFragger within the FragPipe graphical user interface, version 20.0 (https://fragpipe.nesvilab.org/) (75). Data were searched against a human proteome sequence database downloaded from UniProt (date: 20210826) and the sequences for TcdB (P18177) and DT (P00588). This database comprises 20,428 sequences plus their corresponding reverse sequences as decoys and includes common contaminants (76) and various epitope tag sequences, for a total search space of 40,856 sequences. MSFragger search parameters and specifications follow those outlined by Schreiber et al. (2024)(74).

### Interactome analysis and visualization

Statistical analysis of the MSFragger output files was performed using an online implementation of SAINTexpress version 2.5.0 (https://reprint-apms.org) (44). SAINT is a method to rigorously detect proteins enriched by the bait compared to the enzyme-alone control (77). Proteins are considered high-confidence proximal interactors if they have a BFDR ≤0.01 (44). High confidence interactors, determined from SAINTexpress, were input into the GO statistics tool g:Profiler for functional profiling (78). This tool calculates statistical over-representation of molecular functions, biological processes, cellular components, and cellular reaction pathways. Data visualizations of GO enrichment were prepared in the R environment (v4.2.1) (79). We used the Dot Plot Generator and Specificity analysis in ProHits-viz (https://prohits-viz.org) to display the relative abundance of high-confidence interactors and analyze the specificity of high-confidence interactors in the data set. The specificity score is calculated as fold-enrichment, or the abundance of a bait/condition relative to the average abundance across baits/conditions. The Venn diagram was created using FunRich version 3.1.3 (80).

## Data Availability

Mass spectrometry data have been deposited in the Mass Spectrometry Interactive Virtual Environment (MassIVE; http://massive.ucsd.edu) under MassIVE ID MSV000096003.

## SUPPLEMENTAL MATERIAL

Table S1 mbio.03336-24-s0001.xlsx Mapping the interactome of the TcdB glucosyltransferase domain with miniTurbo (related to Fig. 2 and 3).

Table S2 mbio.03336-24-s0002.xlsx Mapping the cell surface interactome of TcdB with recombinant protein on HEK293T cells (related to Fig. 4).

Table S3 mbio.03336-24-s0003.xlsx Mapping the cell surface interactome of TcdB with recombinant proteins on 18Co cells (related to Table 1).

Table S4 mbio.03336-24-s0004.xlsx dtR-TurboID fusion cell surface labeling on ARN8 cells (related to Fig. 5).

ASM does not own the copyrights to Supplemental Material that may be linked to, or accessed through, an article. The authors have granted ASM a non-exclusive, world-wide license to publish the Supplemental Material files. Please contact the corresponding author directly for reuse.
